# An update on reactive astrocytes in chronic pain

**DOI:** 10.1186/s12974-019-1524-2

**Published:** 2019-07-09

**Authors:** Ting Li, Xuhui Chen, Chuanhan Zhang, Yue Zhang, Wenlong Yao

**Affiliations:** Department of Anesthesiology, Tongji Hospital, Tongji Medical College, Huazhong University of Science and Technology, Wuhan, 430030 China

**Keywords:** Reactive astrocytes, A1 astrocytes, A2 astrocytes, Cortical astrocytes, Chronic pain

## Abstract

Chronic pain is a critical clinical problem with an increasing prevalence. However, there are limited effective prevention measures and treatments for chronic pain. Astrocytes are the most abundant glial cells in the central nervous system and play important roles in both physiological and pathological conditions. Over the past few decades, a growing body of evidence indicates that astrocytes are involved in the regulation of chronic pain. Recently, reactive astrocytes were further classified into A1 astrocytes and A2 astrocytes according to their functions. After nerve injury, A1 astrocytes can secrete neurotoxins that induce rapid death of neurons and oligodendrocytes, whereas A2 astrocytes promote neuronal survival and tissue repair. These findings can well explain the dual effects of reactive astrocytes in central nervous injury and diseases. In this review, we will summarise the (1) changes in the morphology and function of astrocytes after noxious stimulation and nerve injury, (2) molecular regulators and signalling mechanisms involved in the activation of astrocytes and chronic pain, (3) the role of spinal and cortical astrocyte activation in chronic pain, and (4) the roles of different subtypes of reactive astrocytes (A1 and A2 phenotypes) in nerve injury that is associated with chronic pain. This review provides updated information on the role of astrocytes in the regulation of chronic pain. In particular, we discuss recent findings about A1 and A2 subtypes of reactive astrocytes and make several suggestions for potential therapeutic targets for chronic pain.

## Background

Pain is an unpleasant sensory and emotional experience associated with actual or potential tissue damage. Pain that lasts more than 3 months is defined as chronic or pathological pain, which is characterised by spontaneous pain, allodynia (pain in response to normally non-painful stimuli), and hyperalgesia (an increased sensitivity to painful stimuli) [1]. Whereas acute pain plays an important protective and survival role via avoidance of harmful stimuli, chronic pain has no clear biological benefits. Chronic pain can be caused by variable noxious stimulation such as major surgery, arthritis, cancer, and nerve injury [2]. As a major health problem, chronic pain affects one third of Americans and costs the US economy $635 billion a year [3], and the prevalence rate of chronic pain is increasing globally every year. However, there are limited effective prevention measures and treatments for chronic pain. To develop a strategy that can inhibit the generation and maintenance of chronic pain, it is necessary to better understand the underlying molecular and cellular mechanisms.

Pain has long been viewed from the “neural centre” perspective, which holds that spinal neuronal pathways regulate “normal” pain signals that become hyperactive during chronic pain [4]. However, in recent years, it has been suggested that spinal glial cells, especially astrocytes, are also involved in the regulation of pain [5, 6].

Astrocytes, as the most abundant cell type in the central nervous system (CNS), play vital roles in maintaining CNS homeostasis. However, after noxious stimulation and nerve injury, the phenotype, functions, and gene expression of astrocytes can undergo a significant change, known as reactive astrogliosis [7]. During this process, naïve astrocytes differentiate into different subsets, including reactive astrocytes and scar-forming astrocytes. Reactive astrocytes can be divided into toxic A1 astrocytes, which induce rapid death of neurons and oligodendrocytes, and neuroprotective A2 astrocytes, which promote neuronal survival and tissue repair [8, 9]. Reactive astrogliosis can increase neuroprotection and nutritional support for damaged neurons. Furthermore, activated astrocytes can reconstruct the damaged blood–brain barrier (BBB) and limit the infiltration of peripheral leukocytes [7, 10]. Thus, astrogliosis is an initial defence mechanism for repairing damage.

However, astrogliosis can also cause some adverse effects [11]. Activated astrocytes may encourage the development and maintenance of chronic pain by releasing signalling molecules [2, 12]. In addition, recent studies have shown that activated astrocytes in brain regions related to emotion regulation (the primary somatosensory (S1) cortex, anterior cingulate cortex (ACC), medial prefrontal cortex, and hippocampus) are associated with emotional dysfunction under chronic pain states [5, 13–15]. Therefore, it is necessary to explore the role and mechanisms of spinal reactive astrocytes in chronic pain, as well as the role of cortical reactive astrocytes in pain and pain-related mood disorders.

## Astrocyte functions in the CNS

**Fig. 1:**
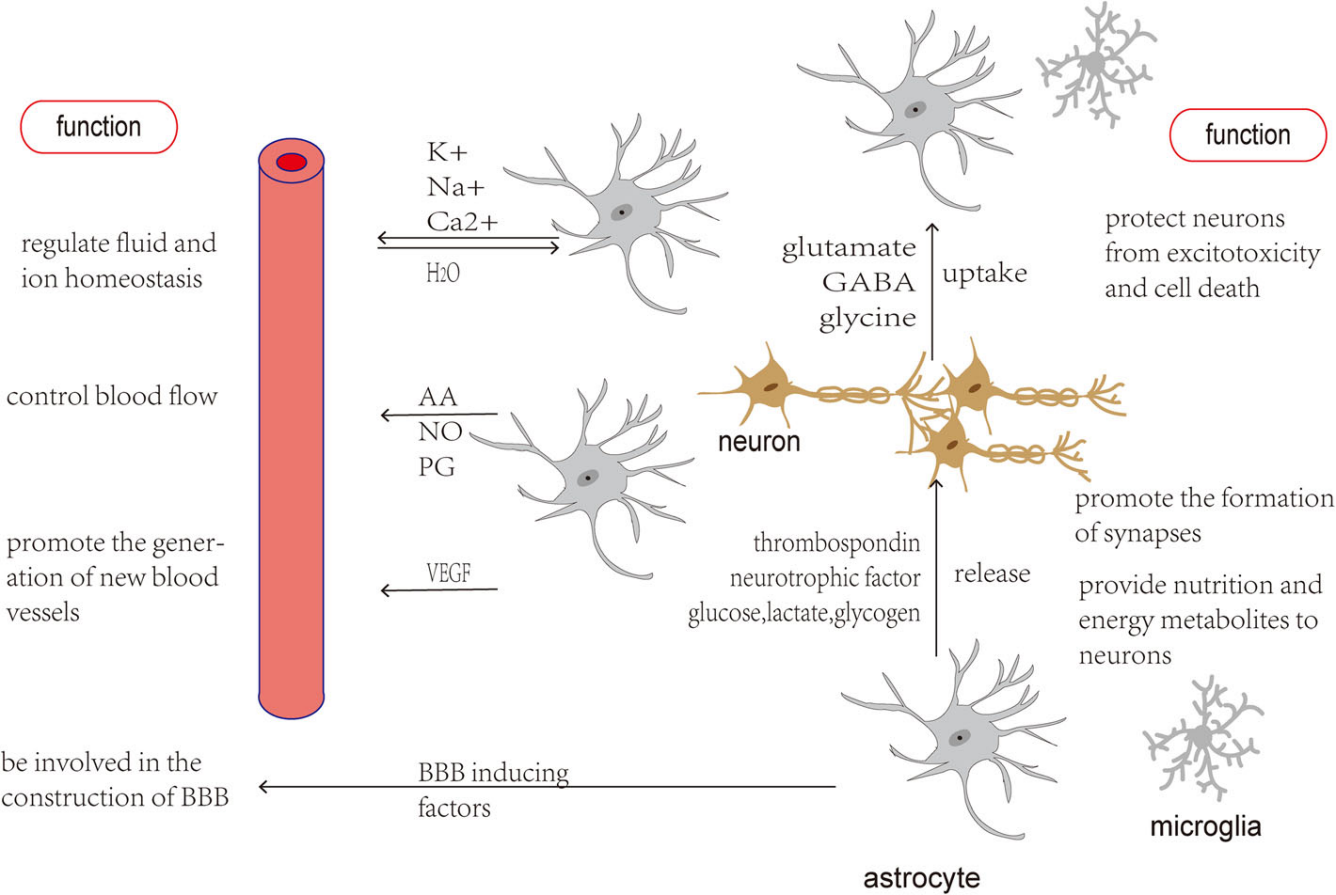
Astrocyte functions in the CNS. Astrocytes play significant roles in the CNS physiology. AA, arachidonic acid; NO, nitric oxide; PG, prostaglandin; VEGF, vascular endothelial growth factor; GABA, γ-aminobutyric acid; BBB, blood–brain barrier

Neural circuits in the CNS are composed of a variety of cell types, including neurons and glial cells. Glial cells in the CNS are composed of three major groups, as follows: microglia, astrocytes, and oligodendrocytes [6]. Astrocytes play a regulatory role in the physiology and pathology of CNS (Fig. 1). For example, astrocytes regulate fluid and ion homeostasis, control blood flow, promote the generation of new blood vessels, protect neurons from excitotoxicity injury and cell death, promote the formation of synapses, provide nutrition and energy metabolites to neurons, and are involved in the construction of BBB [16]. Furthermore, astrocytes modulate microglial phenotypes and phagocytosis through astrocyte-microglia crosstalk and regulate excitatory synaptic transmission through astrocyte-neuron interactions [17, 18].

## Changes in astrocyte morphology and function after noxious stimulation and nerve injury

**Fig. 2:**
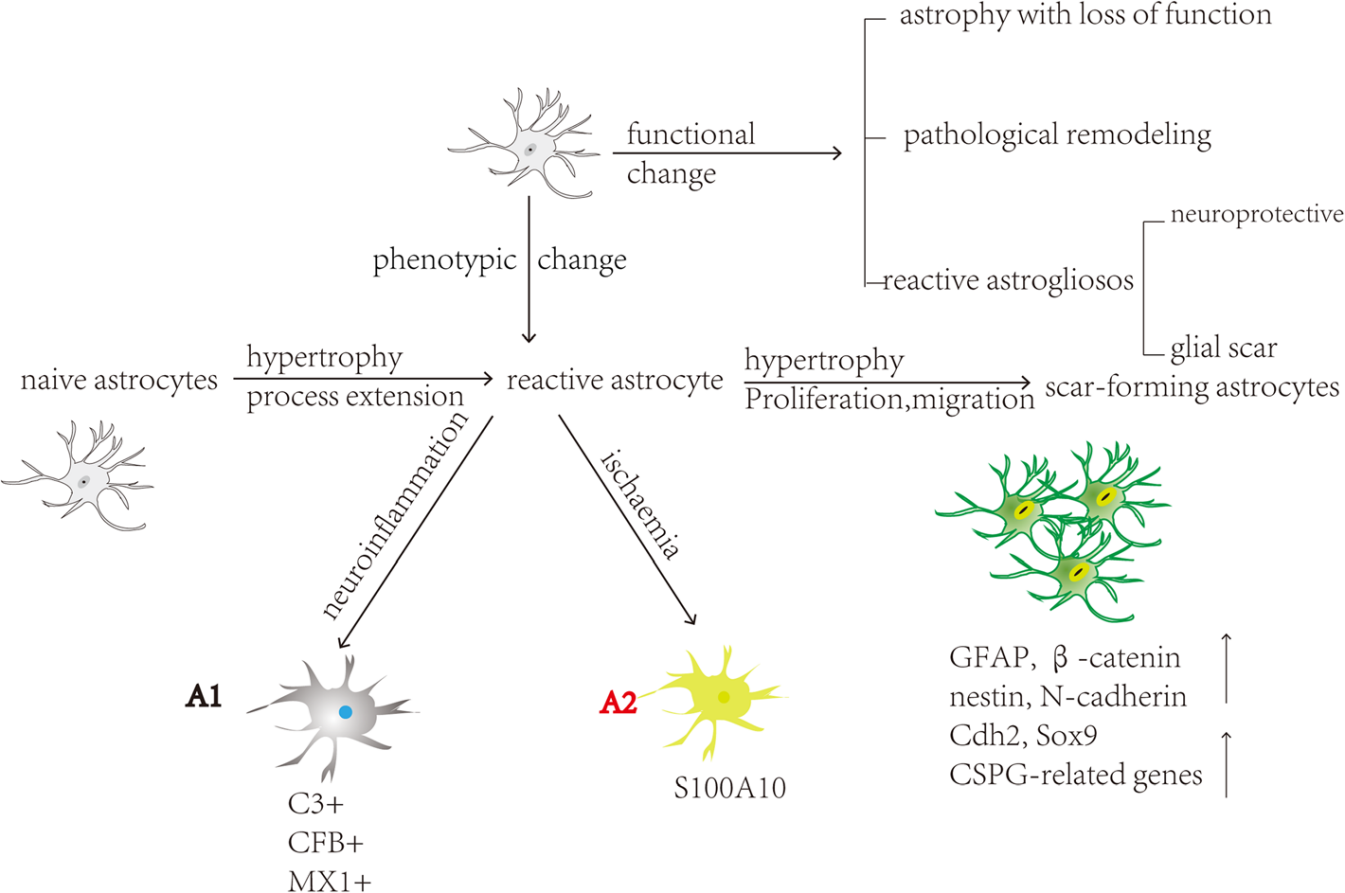
Changes in the morphology and function of astrocytes after noxious stimulation and nerve injury. Based on phenotypic changes of astrocytes, astrocyte can be divided into reactive and scar-forming astrocyte, reactive astrocyte can be further classified by A1 and A2 astrocyte. Based on functional changes of astrocyte, astrocytopathy can be divided into astrocytes atrophy with loss of function, pathological remodelling of astrocytes and reactive astrogliosis based on functional cellular responses

Astrocytes exhibit variable morphological and functional alterations following noxious stimulation and injuries (Fig. 2), including (1) morphological changes (e.g. hypertrophy), (2) proliferation, (3) gene expression changes, (4) significant molecular changes, and (5) functional changes.

### Phenotypic astrocyte changes: reactive and scar-forming astrocytes

After noxious stimulation and nerve injury, astrocytes undergo a series of phenotypic and functional changes in a process called reactive astrogliosis. During this process, naïve astrocytes experience typical changes such as process extension, hypertrophy, and increased GFAP expression, which form the phenotypic characteristics of reactive astrocytes. Thereafter, reactive astrocytes proliferate, migrate, and transform into scar-forming astrocytes [7] (Fig. 2). Both reactive and scar-forming astrocytes highly express astrocyte marker proteins (GFAP, β-catenin, nestin, and N-cadherin), but they have their own marker genes. Reactive astrocyte marker genes include matrix metalloprotease (MMP)2, Plaur, MMP13, Axin2, Nes, and Ctnnb1 and scar-forming astrocyte marker genes include Cdh2, Sox9, and chondroitin sulphate proteoglycan-related genes such as Csgalnact1, Chst11, Pcanwas, Acan, and Slit2 [7, 19, 20]. These marker genes, combined with morphological characteristics, can be used to determine the astrocyte phenotype.

### Classification of reactive astrocytes: A1 and A2 astrocytes

Recently, it has been proposed that neuroinflammation and ischemia induced two different types of reactive astrocytes, which called A1 reactive astrocytes and A2 reactive astrocytes respectively (Fig. 2) [8].

A1 astrocytes, induced by neuroinflammation, secrete neurotoxins that induce rapid death of neurons and oligodendrocytes; however, A2 astrocytes, induced by ischaemia, promote neuronal survival and tissue repair [8]. These two types of reactive astrocytes can be identified according to their individual genetic expressions. Complement 3 (C3), CFB, and MX1S are the most characteristic and significantly upregulated genes in A1 astrocytes and are not expressed in A2 astrocytes; thus, these can be used as specific markers of A1 astrocytes [8]. The S100 protein family member S100A10 has been identified as specific markers of A2 astrocytes [21]. Activated microglia induce the transformation of naïve astrocytes into A1 astrocytes by releasing Il-1α, TNF, and C1q cytokines, each of which is essential for inducing A1 astrocytes [8]. However, milk fat globule epidermal growth factor 8 (MFG-E8) regulates A1/A2 astrocytic conversion through upregulation of the PI3K-Akt pathways and downregulation of the NF-κB pathways [22].

A1 astrocytes lose many normal functions such as the promotion of neuronal survival and outgrowth; furthermore, A1 astrocytes induce fewer and weaker synapses than healthy naïve astrocytes [8]. A1 astrocytes have also been detected in a variety of human neurodegenerative diseases. For example, in Alzheimer’s disease, it has been reported that nearly 60% of GFAP-positive astrocytes in the prefrontal cortex are C3-positive. This indicates that A1 astrocytes may encourage the development of neurodegenerative diseases. By contrast, A2 astrocytes exert neuroprotective and repair tissue effects by secreting several trophic factors. The A2 astrocyte-related gene S100A10 is essential for cell proliferation, membrane repair, and inhibition of cell apoptosis [8]. Moreover, A2 astrocytes promote the expression of anti-inflammatory cytokine TGFβ, which participates in synaptogenesis and plays a neuroprotective role [22].

### Functional astrocyte changes: astrocytopathy

Distinct astrocyte pathological changes may emerge sequentially or coexist during the development of neurological diseases. Astrocytopathy can be classified into astrocyte atrophy with a loss of function, pathological remodelling of astrocytes and reactive astrogliosis based on functional cellular responses (Fig. 2) [23]. Atrophic astrocytes characterised by a loss of function contribute to the pathological progression of various neurological disorders such as epilepsy, schizophrenia and Alzheimer’s disease [24]. Pathological remodelling of astrocytes may be responsible for brain homeostasis disorders, such as the severe white matter encephalopathy seen in Alexander’s disease [25]. Astrogliosis is a complex multifactorial process that can occur after spinal cord injury. During this process, reactive astrocytes that have neuroprotective properties coexist with scar-forming astrocytes that inhibit axonal regeneration as well as functional recovery.

## Molecular regulators and signalling mechanisms involved in the activation of astrocytes and chronic pain

**Fig. 3:**
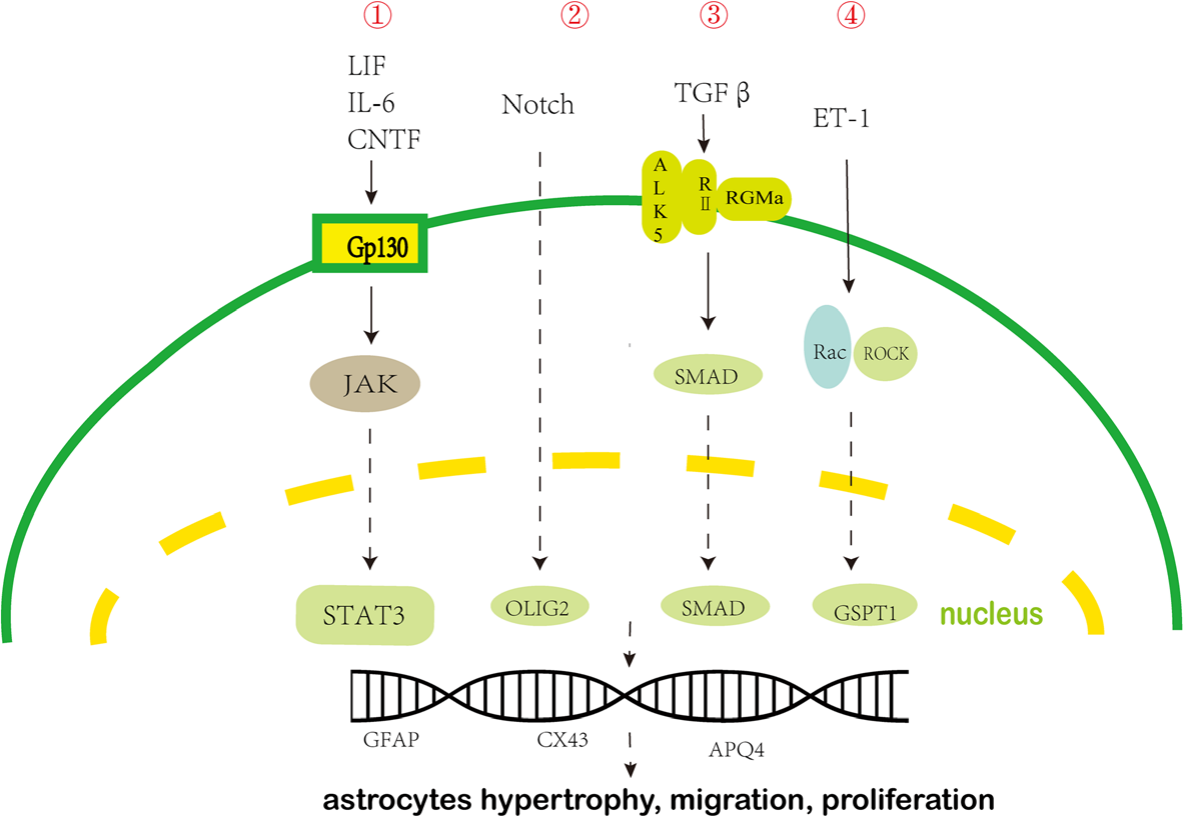
Molecular regulators and signalling mechanisms involved in the activation of astrocytes. The transformation of astrocytes from normal to reactive phenotypes involves a variety of intercellular and intracellular signalling mechanisms that trigger and maintain astrocytes reactivity.① Gp130-JAK-STAT3 signalling pathway. ② Notch-OLIG2 signalling pathway. ③ TGFβ-RGMa-SMAD signalling pathway. ④ Rac-GSPT1 signalling pathway. These signalling pathways regulate the expression of some genes that characterise reactive astrocytes, such as the genes that encode GFAP, CX43, and AQP4, and thus contribute to reactive astrogliosis. Furthermore, multiple signalling molecules in these signalling pathways promote the maintenance and development of chronic pain. IL, interleukin; LIF, leukaemia inhibitory factor; CNTF, ciliary neurotrophic factor; TGF-β, transforming growth factor-β; TNF-α, tumour necrosis factor-α; ET-1, endothelin-1; STAT3, signal transducer and activator of transcription 3; OLIG2, oligodendrocyte transcription factor 2; SMAD, Sma- and Mad-related protein; ROCK, rho associated kinase; GFAP, glial fibrillary acidic protein; CX43, connexin43; RII, type II receptor; ALK5, activatin-like kinase 5; JAK, janus kinase. GSPT1, G1 to S phase transition 1; RGMa, repulsive guidance molecule a

The transformation of astrocytes from normal to reactive phenotypes involves a variety of intercellular and intracellular signalling pathways (Fig. 3) [11]. Signalling molecules that activate naïve astrocytes can be released by many cell types, including neurons and glial cells such as microglia, oligodendrocytes, astrocytes, and inflammatory cells [23].

The signalling molecules involved in the phenotypic transformation of astrocytes include pro-inflammatory cytokines (IL-1β, TNF-α, and IL-6), gene transcription factors (signal transducer and activator of transcription 3 (STAT3), extracellular signal-regulated kinase 1/2 (ERK1/2), oligodendrocyte transcription factor 2 (OLIG2), Sma- and Mad-related protein (SMAD), and G1 to S phase transition 1 (GSPT1)), and proteins (GFAP, connexins, and aquaporin 4 (AQP4)) [26]. Interestingly, these signalling molecules are not only involved in the activation of astrocytes, but also in the development of chronic pain; however, it is unclear whether their role in the development of chronic pain is mediated by the regulation of astrocyte activation.

### Gp130-JAK-STAT3 signalling pathway

Activation of Gp130 receptors by cytokines such as ciliary neurotrophic factor, IL-6, and leukaemia inhibitory factor (LIF) causes phosphorylation of Janus kinase 2 (JAK2). This causes STAT3 to translocate to the nucleus and affect the transcription of certain genes transcription including GFAP, AQP4, connexins, and inflammation-related genes such as nitric oxide synthase 2 (NOS2) (Fig. 3) [27]. In addition, inhibition of astrocytic STAT3 reduces the proliferation and migration of astrocytes after spinal cord injury [26]. Therefore, it is likely that the Gp130-JAK-STAT3 signalling pathway mediates astrocytic proliferation, hypertrophy, migration, and glial scar formation. In a spinal nerve injury model of neuropathic pain, the astrocytic JAK-STAT3 signalling pathway was found to be critical for astrocyte proliferation and maintenance of neuropathic pain [28]. Furthermore, inhibition of astrocyte proliferation by inhibitors of JAK-STAT3 signalling has been found to relieve tactile allodynia induced by spinal nerve injury [28]. This indicates that JAK-STAT3 signalling pathway contributes to the development of neuropathic pain by regulating astrocyte activation.

### Notch-OLIG2 signalling pathway

The conditioned ablation of OLIG2 in astrocytes in one study revealed that OLIG2 is indispensable for the proliferation of reactive astrocytes [29]. Notch, as the upstream signalling molecule of OLIG2, promotes the translocation of OLIG2 into the nucleus of reactive astrocytes [30]. γ-secretase inhibitor (GSI) has been found to inhibit Notch signalling and decrease nuclear-translocation of OLIG2, thereby significantly reducing the proliferation of reactive astrocytes [31]. In a sciatic nerve chronic constriction injury-induced neuropathic pain model, Bertozzi et al. found that pharmacological inhibition of the mRNA expression of astrocyte activation markers (such as GFAP and OLIG2) relieved chronic constriction injury-induced mechanical hyperalgesia. [32] These observations suggest that Notch-OLIG2 signalling plays a pivotal role in astrocyte proliferation and neuropathic pain.

### TGFβ-RGMa-SMAD signalling pathway

TGFβ is a key regulator that initiates reactive astrogliosis and glial scar formation. TGFβ is rapidly upregulated after CNS injury and then activates the SMAD family of transcription factors in astrocytes (Fig. 3) [26]. TGFβ activates the GFAP promoter, which increases vimentin, actin, and GFAP expression in astrocytes via the TGFβ-SMAD3 signalling pathway; besides, TGF-β signalling through SMAD significantly contributes to scar formation after noxious injury and delays nerve recovery [33]. This indicates that astrocytic the TGFβ-SMAD3 signalling pathway plays a key role in the activation of astrocytes.

Repulsive guidance molecule a (RGMa) is a newly discovered membrane protein that mediates reactive astrogliosis and glial scar formation by regulating the TGFβ1-SMAD2/3 pathway. Knockdown of RGMa reduces TGFβ1-induced reactive astrogliosis and glial scar formation and promotes functional recovery after stroke in a rat middle cerebral artery occlusion/reperfusion model [34]. It has also been reported that RGMa is upregulated after rat and human spinal cord injury and that an RGMa antibody attenuated the associated neuropathic pain [35]. These results are indicative of the therapeutic potential of targeting the TGFβ-RGMa-SMAD signalling pathway to reduce neuropathic pain.

### Rac-GSPT1 signalling pathway

Activation of receptors for ET-1, thrombin, and TGF-β activate signalling mechanisms mediated by Rho family proteins, such as RhoA and Rac1 that then induce astrocyte activation [26, 36]. The major downstream effector of Rho is Rho-associated protein kinase (ROCK), and ROCK inhibition induces rapid and reversible stellation of cultured astrocytes and an increase in their migratory activity [36]. Many studies have shown that activation of the RhoA/ROCK signalling pathway contributes to the development and maintenance of inflammatory pain, neuropathic pain, and bone cancer pain [37, 38]. Therefore, the Rho family and its effector, ROCK, are not only involved in the activation of astrocytes, but also in the development of chronic pain. The Rac- GSPT1 (G1 to S phase transition 1) signalling pathway in astrocytes is a novel candidate for reactive astrogliosis and glial scar formation. Previous studies have reported that Rac1-KO and Rac-KD mice showed better functional recovery and reduced astrogliosis and glial scar formation after CNS injury compared with control mice. GSPT1 protein, which is a novel downstream target of Rac1, facilitates cell proliferation by accelerating the transformation of the G1 to S phase; GSPT1-KD astrocytes have been reported to exhibit a delayed cell cycle [39]. However, it is not clear whether GSPT1 is associated with chronic pain.

## The role of reactive astrocytes in chronic pain

### The dual role of reactive astrocytes

**Fig. 4:**
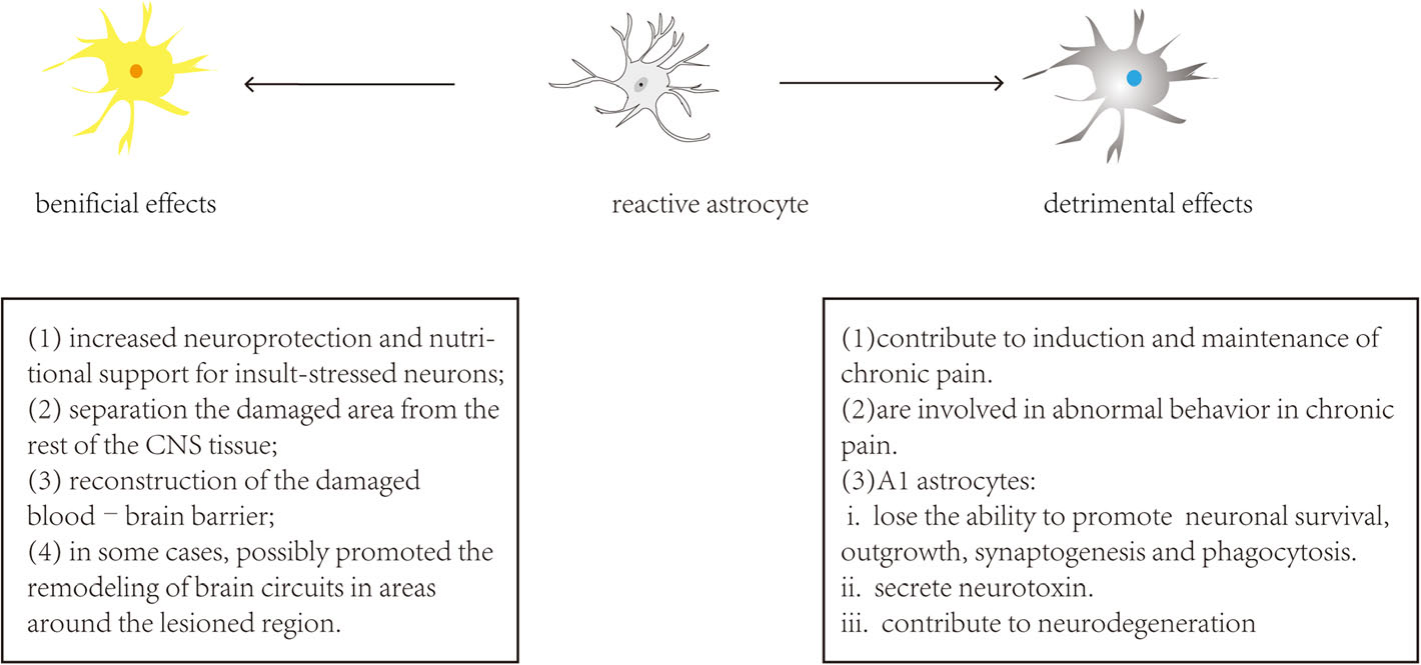
Activation of astrocytes has dual effects. Astrogliosis is a defence mechanism for repairing initial damage, but it can also have adverse effects

Astrogliosis is a double-edged sword (Fig. 4). As a defence mechanism, Reactive astrogliosis can increase neuroprotection and nutritional support for insult-stressed neurons. Besides, activated astrocytes can reconstruct the damaged blood–brain barrier and limit the infiltration of peripheral leukocytes [23]. Reactive astrocytes, which are a transitional form of astrocytes in reactive astrogliosis, play an important role in tissue repair and neuroprotection in the subacute stage of spinal cord [7]. For example, it has been reported that the β-catenin–MMP pathway is involved in the migration of astrocytes and tissue repair after noxious stimulation and nerve injuries. TGF-β signalling that participates in astrogliosis promotes neuronal survival as well as axonal regeneration [40, 41].

A2 astrocytes exert neuroprotective and repair tissue effects by secreting several of trophic factors. A2 astrocyte-related gene S100A10 is essential for cell proliferation, membrane repair, and inhibition of cell apoptosis [8]. Besides, A2 astrocytes promote the expression of the anti-inflammatory cytokine TGFβ, which participates in synaptogenesis and plays a neuroprotective role [22]. Besides, Myer et al. have shown that reactive astrocytes play an important role in protecting nerve tissue and limiting inflammation following moderate focal brain injury [42].

Although the present evidence suggests that reactive astrocytes have important neuroprotective and repair roles in the initial stage of nerve injuries, their inhibitory effects on functional recovery after injury are undeniable (Fig. 4). In particular, accumulating evidence indicates reactive astrocytes contribute to the persistent and development of chronic pain [4].

Activation of microglia and astrocyte has been observed in central neuropathic pain models such as spinal cord injury, spinal cord infection models, and peripheral neuropathic pain models such as peripheral nerve injury [43]. In addition, astrocytes are more closely associated with chronic pain behaviour and synapse after nerve damage and are more persistent than microglia reaction [12]. During the development of pain, microglia responses are typically early and transiently, while astrocyte activation later and last longer than microglia. Pharmacologically, minocycline can be used as microglia inhibitors to prevent the induction of pathological pain, but it usually does not reverse the abnormal pain and hyperalgesia established after nerve injury/inflammation [43, 44]. However, Intrathecal injection of the astrocytes inhibitors, such as valerine, fluorocitrate, and l-1-amino-hexanedioic acid, could effectively reverse mechanical allodynia and reduce the maintenance of abnormal pain and hyperalgesia in the pathologic pain model [43]. These studies indicate microglia may contribute to the initiation of mechanical allodynia, while astrocytes may be responsible for their maintenance.

Liddelow et al. speculate that A1 reactive astrocytes may be neurotoxic, while A2 reactive astrocytes may be neuroprotective [8]. These findings can well explain the dual effects of reactive astrocytes in central nervous injury and diseases. However, it is unclear whether reactive astrocytes have a bilateral role in chronic pain. We guess that different subtypes of reactive astrocytes might play dual roles in chronic pain associated with CNS injury.

### Intracellular kinases, channels, receptors, pro-inflammatory cytokines, chemokines, and proteases in astrocytes involved in pain regulation

The expression of multiple signalling molecules and receptors in astrocytes changes in chronic pain states, facilitating or inhibiting chronic pain. These molecules include intracellular kinases, pro-inflammatory cytokine, chemokine, proteases, etc. (Table 1).

**Table 1:** Signalling molecules and receptors in astrocytes involved in pain regulation

Pro-inflammatory cytokines	TNF-α
	IL-1β
	IL-6
Chemokines	CCL-2, CCL-3, CCL-5
	CXCL-1
Receptors	TLR-4
	TLR-2
Connexins	Cx30, Cx43
Intracellular kinases	MAPK:ERK1/2, P38, JNK
Proteases	tPA
	MMP

#### Intracellular kinases

The mitogen-activated protein kinase (MAPK) family consists of p38, ERK1/2, and JNK. Several studies have shown that inhibition of JNK, p38, and ERK can effectively reduce neuropathic pain in various pain models [45–47]. In one study, application of MAPK and ERK inhibitors in the spinal cord inhibited the late-stage activation of ERK and reversed mechanical allodynia, which indicates that astrocytic ERK plays a role in the maintenance of neuropathic pain [47]. The induction of p-ERK in glial cells after injury is highly variable; p-ERK is induced in spinal microglia in the acute phase and induced in astrocytes in the late phase after nerve injury [45, 47].

Recently, it has been demonstrated that JNK in spinal cord astrocytes plays an important role in neuropathic pain after nerve injury. Spinal injection of a JNK inhibitor has been found to relieve neuropathic pain after nerve injury and diabetes [48]. CXCL1 upregulation in astrocytes after JNK activation promotes central sensitisation and neuropathic pain; furthermore, spinal inhibition of CXCL1 can relieve nerve injury-induced pain hypersensitivity [17, 49]. This indicates that activation of the JNK pathway in spinal cord astrocytes and subsequent upregulation of CXCL1 play an important role in neuropathic pain.

#### Regulation of receptors and channels in astrocytes

Toll-like receptor 4 (TLR-4) is highly expressed in microglia and astrocytes. In mice models of chronic constriction injury of the sciatic nerve, TLR-4 and chaperone CD14 gene expression were found to increase after spinal cord nerve transection; furthermore, chronic constriction injury-induced thermal hyperalgesia and mechanical allodynia were attenuated by intrathecal injection of the TLR-4 antagonist [50].

Several studies have demonstrated that toll-like receptor 2 (TLR2) contributes to persistent pain via neuroinflammatory mechanisms. Nerve transection injury results in increased TLR2 immunoreactivity in the spinal cord, along with increased microglial nicotinamide adenine dinucleotide phosphate-oxidase, which induces an inflammatory response [51]. After nerve injury-induced microglial and astrocytic activation in TLR-2-knockout mice, there is a reduction in gene expression of pro-inflammatory factors (such as IL-1β and TNF-α) in the spinal cord and in the hypersensitivity to pain [52]. Therefore, TLR-4 and TLR-2 of astrocytes are involved in pain regulation.

Astrocytes characteristically form gap junction-coupled networks, which leads to the transmission of Ca^2+^ signals through the networks [53]. Connexins (CX) are the main structural components of gap junctions, and Cx30 and Cx43 are specifically expressed in astrocytes [54]. Interestingly, the expression of Cx43 increases significantly in response to spinal cord injury and facial nerve lesions [12], which suggests that CX plays an important role in chronic pain. The suppression of gap junction function by carbenoxolone (CBX), which is a gap junction decoupler, attenuates the inflammatory response and neuropathic pain [6]. In particular, intrathecal injection of CBX reduces mechanical allodynia induced in the rat contralateral paw by sciatic nerve inflammation [55], which suggests that astrocyte gap junctions contribute to the spread of pain beyond the injury site.

#### Regulation of pro-inflammatory cytokines, chemokines, and proteases in microglia and astrocytes

Activated microglia and astrocytes release various pro-inflammatory cytokines and chemokines following injury. IL-1β, IL-6, and TNF-α are the most studied pro-inflammatory mediators. They are not only involved in astrocyte-microglia crosstalk but have also been related to inflammatory pain, bone cancer pain, and neuropathic pain [6, 43, 56].

IL-1β is upregulated in spinal microglia and astrocytes following peripheral nerve injury. Accumulating evidence has implicated IL-1β to be involved in pain sensitisation [43, 56]. Inflammation, neuropathy, and cancer pain are reduced after intrathecal injection of IL-1 receptor antagonists or neutralising antibodies to inhibit IL-1 signalling in the spinal cord [12]. In peripheral nerve injury models, mechanical hypersensitivity was reported to be significantly reduced in mice lacking IL-1β and IL-1α [57]. IL-1β also directly sensitises heat and chemical-sensitive cation channels in the transient receptor potential cation channel subfamily V member 1 (TRPV1) [56].

TNF-α is primarily produced by microglia and plays an important role in the initiation and development of persistent pain and central sensitisation; its role in regulation of peripheral sensitisation has also been demonstrated [6]. The intrathecal injection of exogenous TNF has been found to result in both mechanical and thermal hypersensitivity, while intrathecal injection of anti-TNF antibodies before peripheral nerve injury prevents the development of neuropathic pain in several neuropathic pain models [58]. This indicates that these cytokines contribute to the development of chronic pain.

Chemokines are widespread in the blood and immune system. Recent studies have found that chemokines are also abundant in the CNS and are expressed in glial cells, particularly in astrocytes and neurons [56]. Besides, chemokines contribute to development of neuropathic pain and inflammatory pain by inducing neuroinflammatory responses. The intrathecal injection of CCL2, CCL3, and CCL5 can elicit pain behaviours, and these effects may be caused by the activation of chemokine receptors in the dorsal root ganglia [56].

After peripheral nerve injury, CCL2 which is expressed and released by spinal astrocytes has been found to significantly increase and to promote the maintenance and development of neuropathic pain and is involved in pain regulation via direct action to sensory neurons [1]. CCL2 has been found in astrocytes in the brain after demyelinating lesions, mechanical injury, and focal cerebral ischaemia [12]. CCL2 has been found to rapidly increase AMPA- and NMDA-induced inward currents in spinal dorsal horn neurons; this is indicative of increased glutamatergic synaptic transmission, which is closely related to central sensitisation and hyperalgesia [1, 12]. In addition to its action on neurons, CCL2 released by astrocytes may also induce the proliferation and migration of microglia in the spinal cord, thereby further enhancing pain [1]. In recent years, the key role of peripheral CCL3 in neuropathic pain has also been demonstrated. For example, one study found that CCL3-induced neuropathic pain is evoked partly through IL-1β upregulation [56].

CXCL1 is also an important chemokine involved in the interaction between neurons and astrocytes [56]. In models of spinal nerve ligation, the expressions of CXCL1 and its receptor (CXCR2) were increased in spinal cord astrocytes and neurons, respectively [49]. In this study, intrathecal injection of anti-CXCL1 antibody was found to attenuate spinal nerve ligation-induced pain hypersensitivity, and the CXCR2 antagonist completely blocked the CXCL1-induced heat hyperalgesia. This suggests that CXCL1 and CXCR2 play an important role in neuropathic pain.

Astrocytes express proteases, such as MMPs and tissue-type plasminogen activator (tPA), which are thought to be key in the cleavage of pro-inflammatory cytokines from astrocytes [12]. Nerve injury leads to increased activity of MMP2 and MMP9, which then activate IL-1β and microglia [6]. Furthermore, MMP9 released from neurons has been found to participate in the early stage of neuropathic pain, whereas MMP2 released from astrocytes has been found to be involved in the late stage of neuropathic pain [59].

In one study, Kozai et al. showed that only activated astrocytes expressed tPA after spinal cord injury and that intrathecal injection of tPA inhibitor inhibited mechanical allodynia induced by dorsal root ligation [60]. tPA released from activated astrocytes may affect pain behaviour by activating microglia and regulating neuronal plasticity.

## The role of cortical astrocytes in chronic pain

**Fig. 5:**
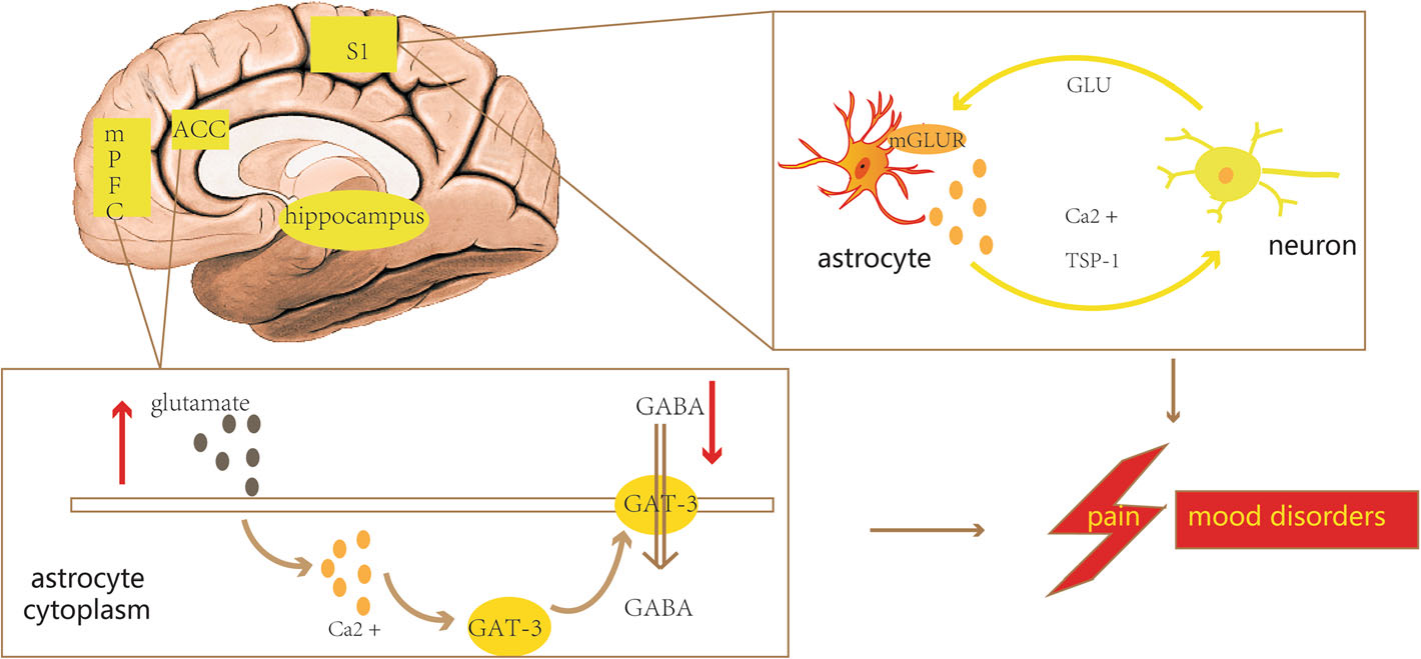
The role of cortical reactive astrocytes in chronic pain. Activated astrocytes in brain regions related to emotion regulation (the S1, ACC, medial prefrontal cortex, and hippocampus) are involved in both pain and pain-related emotional dysfunction. The imbalance between glutamate and GABA due to astrocyte activation in these regions may be one mechanism underlying chronic pain. ACC, anterior cingulate cortex; GAT-3, GABA transporters3; mGluR5, metabotropic glutamate receptor 5; mPFC, the medial prefrontal cortex; S1, somatosensory cortex; TSP-1, thrombospondin 1

In addition to reactive astrocytes around damaged areas, cortical astrocytes also play an important role in chronic pain (Fig. 5). Patients with chronic pain often experience depression, anxiety, insomnia, and other emotional disorders [61]. Recent studies have shown that activated astrocytes in brain regions related to emotion regulation (the S1, ACC, medial prefrontal cortex, and hippocampus) are associated with emotional dysfunction in chronic pain states [14, 62].

### Primary somatosensory cortex

The S1 region plays a role in identifying the intensity and location of pain, and S1 activity is significantly greater in patients with chronic pain than in those without chronic pain [62]. Ishikawa et al. reported that astrocytic activities increased in the ipsilateral S1 following partial sciatic nerve ligation [63]. Activated astrocytes in S1 release a synaptic formation molecule that leads to synaptic remodelling, which in turn leads to chronic pain [13]. It has been reported that rearrangement of the cortical neuronal network induced by S1 astrocytes not only induces abnormal pain in the injured limb, but also in the uninjured contralateral limb, which is called mirror pain [63]. This suggests that activated astrocytes in S1 may be involved in the development of central sensitization.

Kim et al. have shown that sciatic nerve ligation induces immature metabotropic glutamate receptor 5 (mGluR5) signalling in S1 astrocytes, thereby increasing the Ca^2+^ transients in the astrocytes; this then induces Ca^2+^-dependent thrombospondin 1 release and synaptic formation, leading to mechanical hyperalgesia [13]. This phenomenon has also been reported in the medial prefrontal cortex [64]. However, blocking the astrocytic mGluR5-signalling pathway was found to inhibit hyperalgesia [13]. These studies suggest that activated astrocytes in the S1 and medial prefrontal cortex are involved in chronic pain.

### Anterior cingulate cortex

Several studies using a variety of chronic pain models have shown that GFAP expression in the ACC increases under chronic pain [65, 66], which indicates that activated astrocytes in the ACC are involved in chronic pain. It is noteworthy that astrocytes play a key role in regulating the balance between GABA and glutamate. An imbalance between glutamate and GABA due to astrocyte activation in the ACC may be one mechanism underlying chronic pain. For example, sciatic nerve ligation has been found to result in significant increase of glutamate release in the ACC and then increased glutamate was found to modify Ca^2+^ levels in astrocytes; in turn, this induced Ca^2+^-dependent translocation of GABA transporter 3 to astrocyte membranes, which uptake extracellular GABA [66]. Thus, the simultaneous increase in glutamate and decrease in GABA enhances the pain signal. In addition, in this study, Yamashita et al. have also demonstrated that astrocytic activation in the ACC can induce sleep disorders under chronic pain. Similarly, inhibition of astrocyte function in this region can reduce anxiety in patients with chronic pain [15]. Narita et al. have also demonstrated that chronic pain induces anxiety-like behaviour by promoting activation of astrocytes in the ACC [67]. Therefore, the ACC may be involved in regulating the experience of pain and the negative emotions associated with pain.

### Hippocampus

The hippocampus plays an important role in spatial memory and emotional state and is crucial for the development of co-morbid behaviour following nerve damage. Accumulating evidence has indicated that adaptive changes in hippocampal astrocytes are associated with mood disorders in persistent pain states. For example, the numbers of GFAP-positive hippocampal astrocytes were increased in rats undergoing sciatic nerve transection compared with sham-operated rats [61]. Similarly, a rat model of pain hypersensitivity caused by morphine tolerance increased the number of reactive astrocytes in the cingulate cortex and hippocampus [68]. In contrast, antidepressant drugs were found to significantly inhibit hippocampal astrocyte activation and alleviate mechanical hyperalgesia [69]. However, Fiore et al. have shown that hippocampal astrocyte atrophy and reduced GFAP expression is related to pain and mood disorders after nerve injury [70]. This may be due to the different subtypes of activated astrocytes in the hippocampus that play a role in persistent pain and mood disorders.

The relationship between cortical astrocytes and co-morbid behaviours in pain remains to be elucidated. Future studies should focus on whether different subtypes of astrocytes play a role in pain-related affective disorders.

**Fig. 6:**
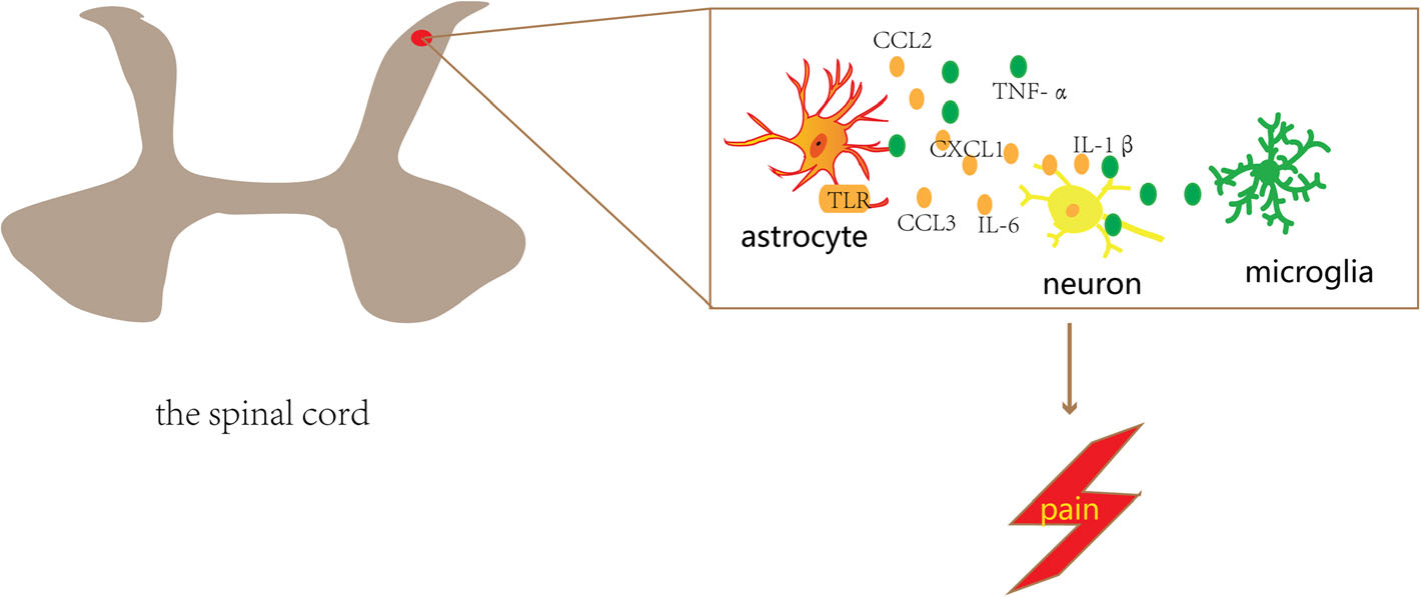
The role of spinal reactive astrocytes in chronic pain. Astrocyte-microglia crosstalk and astrocyte-neuron crosstalk facilitate the development of chronic pain. For example, reactive astrocyte can receive multiple molecular signals from microglia as well as neuron and can secrete several of molecular signals that act on neuron and microglia thereby promoting the development of pain. TLR, toll-like receptor

## Conclusions and future directions

### What is the function of A1 and A2 subtypes of reactive astrocytes in chronic pain?

**Fig. 7:**
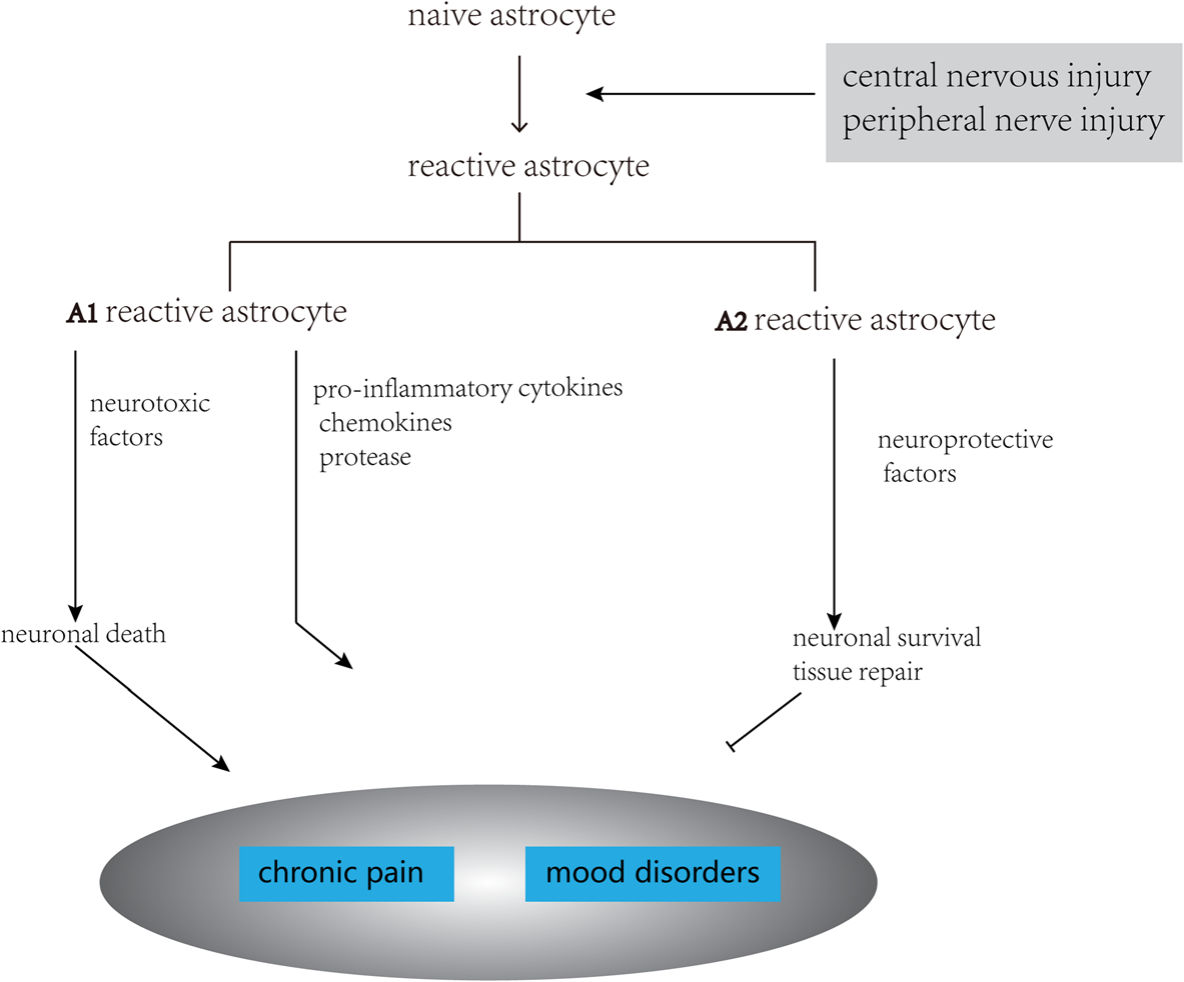
The possible mechanism of A1 and A2 subtypes of reactive astrocytes in chronic pain. A1 reactive astrocytes may facilitate directly the development of chronic pain by releasing some molecules such as pro-inflammatory cytokines, chemokines, and intracellular kinases. It can also indirectly lead to chronic pain by inducing neuronal death. A2 astrocytes may inhibit the progression of chronic pain by secreting neuroprotective factors that promote neuronal survival

The neuroprotective and neurotoxic effects of reactive astrocytes in neurodegenerative diseases and spinal cord injury have been demonstrated in previous studies [42, 71–73]. As described above, A1 astrocytes not only lose many normal functions but also secrete toxic factors that rapidly kill mature oligodendrocytes and neurons. Moreover, A1 astrocytes have been associated with a variety of human neurodegenerative diseases [8]. These studies suggest that the detrimental effect of reactive astrocytes after injury can be attributed to A1 reactive astrocytes. Astrocyte reactivity is a common feature of various diseases, such as neurodegenerative diseases, spinal cord injury, and chronic pain. Currently, a large number of studies have shown that reactive astrocytes contribute to the development of chronic pain (Fig. 6); however, the protective effect of reactive astrocytes on pain is not yet fully understood, and it is unclear whether they have a bilateral role in chronic pain. We can hypothesise that A1 reactive astrocytes may be involved in chronic pain by causing the release of molecules such as pro-inflammatory cytokines, chemokines, and intracellular kinases (Fig. 7).

In contrast, A2 reactive astrocytes may exert neuroprotective effects and repair tissue after injury. For example, activated astrocytes can reconstruct the damaged BBB and limit the infiltration of peripheral leukocytes [42]. Therefore, the beneficial effect of reactive astrocytes after injury can be attributed to A2 reactive astrocytes, which may delay or even prevent the progression of chronic pain.

A recent study reported that spinal microglia showed increased M1 polarisation and decreased M2 polarisation in the development of bone cancer pain [74]. Dehydrocorydaline inhibited the polarisation of spinal microglial cells to the M1 phenotype, increased the number of M2 microglia, and alleviated bone cancer pain [74]. Thus, like microglia, astrocytes in the spinal cord may polarise to A1 and A2 phenotypes and play opposite roles in chronic pain (Fig. 7).

### Conversion of A1 and A2 reactive astrocytes could represent a strategy for the prevention and treatment of chronic pain

Many previous studies demonstrated that intrathecal injection of the astrocytes inhibitors, such as valerine, fluorocitrate, and l-1-amino-hexanedioic acid, effectively reverse mechanical allodynia and reduce the maintenance of abnormal pain and hyperalgesia in a model of pathologic pain [75, 76]. However, reactive astrocytes play a key role in the repair of damaged CNS tissue and positively affect the recovery process after spinal cord injury via STAT3 signalling. Indeed, a mouse model with conditioned knockout of STAT3 from astrocytes showed inhibited astrogliosis and also increased inflammatory infiltration and enlargement of the damaged area after spinal cord injury [77]. Therefore, specific inhibition of A1 reactive astrocytes may represent a potential therapeutic target that is more accurate and has fewer side effects than the direct use of astrocyte inhibitors.

NeuroD1 is an endogenous neurotranscription factor. Zhang et al. have shown that A1 reactive astrocytes can transform into less harmful astrocytes and scar-forming astrocytes can convert into neurons, when NeuroD1 is expressed by reactive astrocytes [78]. Guo et al. have also demonstrated that, following NeuroD1 expression, reactive astrocytes transform into functional neurons in a mouse model of Alzheimer’s disease; the same phenomenon was observed in cultured human cortical astrocytes [79]. Similarly, Chen et al. have reported that when NeuroD1 is highly expressed in astrocytes, the new neurons transformed by astrocytes recovered 40% of the neurons lost in ischaemic injury and restored motor and memory deficits. These studies suggest that A1 astrocytes have the potential to transform to A2 astrocytes even to naïve astrocytes [80]. If A1 astrocytes contribute to the development of chronic pain, then promoting the transformation of A1 into A2 astrocytes even into naïve astrocytes could represent a potential therapeutic strategy to reduce pain.

Blocking the upstream molecules or the downstream targets of A1 reactive astrocytes may also be an effective therapy. Liddelow et al. have shown that activated microglia induce the transformation of naïve astrocytes into A1 astrocytes by releasing Il-1α, TNF, and C1q [8]. Xu et al. have demonstrated that MFG-E8 regulates A1/A2 astrocytic conversion through upregulation of PI3K-Akt pathways and downregulation of NF-κB pathways in vitro [22]. Therefore, specific inhibition of these signalling molecules may alleviate chronic pain. In addition, the A1/A2 astrocytic conversion in vivo remains unknown and deserves further study.

### Whether A1 and A2 astrocytes in the cortex are involved in pain-related mood disorders?

As discussed in this review, reactive astrocytes in brain regions related to emotion regulation have been associated with emotional dysfunction in chronic pain states. It is not yet known whether cortical A1 astrocytes are also involved in pain-related affective disorders. A1 astrocytes may represent a target for the treatment or even prevention of chronic pain and pain-related behaviour changes.

At present, we know that reactive astrocytes include A1 and A2 subtypes, and activated microglia include M1 and M2 subtypes. However, it is possible that there are other glial cell subtypes that play different roles in the development of diseases. Therefore, it is urgent to characterise the different activation states of glial cells.

### The role of cross-talk between astrocytes and neurons in chronic pain

Astrocyte activation can cause dysregulation of glutamate and GABA. This leads to an imbalance of excitatory and inhibitory neuronal inputs, which in turn enhances pain signals. This phenomenon occurs in many brain regions, including the S1, medial prefrontal cortex, and ACC [13, 63]. Like astrocyte-neuron crosstalk in the cortex, the interactions between CXCL2 secreted by astrocytes and CXCR2 expressed by neurons in the spinal cord also contribute to neuropathic pain [49]. Recent work has shown that CXCL1/CXCR2-mediated interactions between astrocytes and neurons in the supraspinal pain regulatory system facilitate chronic pain [17]. Liu et al. have also demonstrated that the CXCL12/CXCR4-mediated crosstalk between astrocytes and neurons maintains neuropathic pain through a central sensitisation mechanism [81]. These studies suggest that cytokines play an important role in the crosstalk between glial cells and neurons. Therefore, the molecular mechanism of this crosstalk in the spinal cord and brain in the context of chronic pain should be further studied.

In conclusion, activation of astrocytes in the spinal cord and cortex plays an important role in the maintenance of chronic pain. It is of great significance to clarify the mechanism of astrocyte activation for the prevention and treatment of chronic pain. The discovery of A1/A2 astrocytes may well explain the dual effects of reactive astrocytes in central nervous injury and diseases. To explore their role in chronic pain and the mechanism of transformation between them may provide new prevention and treatment strategies for chronic pain.

## Data Availability

Not applicable

## Abbreviations

ACC: Anterior cingulate cortex
ALK5: Activatin-like kinase 5
AQP4: Aquaporin 4
BBB: Blood–brain barrier
C3: Complement 3
CBX: Carbenoxolone
CNS: Central nervous system
CX: Connexins
ECM: Extracellular matrix
EGF: Epidermal growth factor
ERK: Extracellular signal-regulated kinase
ERK1: Extracellular signal-regulated kinase 1
FAK: Focal adhesion kinase
FGF2: Fibroblast growth factor-2
GAT-3: GABA transporters3
GFAP: Glial fibrillary acidic protein
GSI: γ-secretase inhibitor
GSPT1: G1 to S phase transition 1
JAK: Janus kinase
JNK: C-Jun N-terminal kinases
MAPK: Mitogen-activated protein kinases
MCP-1: Monocyte chemoattractant protein-1
MFG-E8: Milk fat globule epidermal growth factor 8
MMP: Matrix metalloproteases
mPFC: The medial prefrontal cortex
RGMa: Repulsive guidance molecule a
RII: Type II receptor
ROCK: Rho associated kinase
S1: Somatosensory cortex
SMAD: Sma- and Mad-related protein
STAT3: Signal transducer and activator of transcription 3
TLR: Toll-like receptor
tPA: Tissue-type plasminogen activator
TRPV1: The transient receptor potential cation channel subfamily V member 1
TSP-1: Thrombospondin 1
